# Perioperative use of enteral nutrition with ω-3 polyunsaturated fatty acid in patients with gastric cancer: a meta-analysis

**DOI:** 10.3389/fonc.2024.1488229

**Published:** 2024-10-14

**Authors:** Tingting Fu, Wenjun Hu, Lu Chang, Jingli Duan

**Affiliations:** ^1^ Clinical Trial Center Office, Beijing GoBroad Hospital, Beijing, China; ^2^ Department of Pharmacy, Fengtai District Maternal and Child Health Care Hospital, Beijing, China

**Keywords:** gastric cancer, immuno-enhanced type, enteral nutrition, efficacy, safety

## Abstract

**Objectives:**

To systematically evaluate the efficacy and safety of enteral nutrition with ω-3 polyunsaturated fatty acid preparations or standard enteral nutrition preparations used in patients with gastric cancer during perioperative period, and to provide reference for clinical rational drug use.

**Methods:**

Pubmed, EMbase, The Cochrane Library, CNKI and Wanfang Medical databases were searched by computer to collect relevant literature. The search period was from the establishment of the database to August 1, 2024. Meta-analysis was performed using Revman5.4 software after two researchers independently screened literature, extracted data, and evaluated the risk of bias in included studies.

**Results:**

A total of 20 randomized controlled studies were included. The Meta results showed that there was no statistical difference in mortality between the enteral nutrition with ω-3 polyunsaturated fatty acid group and the control group (RR = 0.46, *P* = 0.17). However, the IEN group demonstrated superior advantages in reducing infection complications (RR = 0.81, *P* = 0.05) and wound infection (RR = 0.61, *P* = 0.04) among gastric cancer patients, as well as improving immune-related indicators (including IgG, IgA, IgM, CD4, and CD4/CD8), inflammation-related markers (including CRP, IL-1β, and IL-6), and nutritional indicators (including Total protein, Albumin, and Transferrin).

**Conclusion:**

enteral nutrition with ω-3 polyunsaturated fatty acid preparation has advantages in the efficacy and safety of perioperative nutritional therapy in patients with gastric cancer, and can be used as a clinical choice. Due to the limited number and quality of included studies, the above conclusions need to be verified by more high-quality studies.

## 1 Introduction

According to the latest estimate of the Global Cancer (GLOBOCAN) statistical report, the annual incidence of gastric cancer in the world in 2020 is 1.089 million people (corresponding to the age-standardized incidence of 11.1 per 100,000 people), ranking fifth among all malignant tumors (1). A study published in 2022 estimated that new cases of stomach cancer worldwide will increase by 62% to 1.77 million cases by 2040 (2). Clinical evidence shows that most gastric cancer patients have serious nutrient absorption disorders and lower immune function than normal patients (3). Continuous malnutrition will reduce the tolerance of gastric cancer patients to chemotherapy, seriously hinder the completion of chemotherapy plan, and affect the therapeutic effect of cancer.

There are two main ways of clinical nutrition support therapy: enteral nutrition (EN) and parenteral nutrition. After surgical operation, patients will have high metabolism and negative nitrogen balance, which will affect their absorption of nutrients and easily lead to malnutrition (4). EN refers to the nutritional support mode that provides nutrients required for human metabolism through the gastrointestinal tract (5). Enteral immune nutrition preparations refer to the addition of glutamine, arginine, ω-3 unsaturated fatty acids, nucleotides and other immunomodulatory nutrients into standard enteral nutrition formulations to improve cellular immune function by affecting the production and release of local or systemic inflammatory factors (6). Studies have shown that immune-enhanced EN preparations containing immune regulatory components of ω-3 polyunsaturated fatty acids are beneficial to patients undergoing surgery for head and neck tumors and upper digestive tract tumors by reducing the risk of infection, improving immune and inflammation-related indicators, maintaining body mass and reducing hospital stay (7, 8).

The systematic evaluation of the clinical value of ω-3 polyunsaturated fatty acid nutrition drugs is an urgent requirement to promote the rational use of nutrition drugs in clinic, and it is also an effective way to enhance the therapeutic effect of diseases and improve the health of people. However, up to now, there is still a lack of high-quality systematic reviews on the efficacy evaluation and analysis of ω-3 polyunsaturated fatty acids, especially the lack of systematic reviews on the safety and effectiveness of perioperative use of ω-3 polyunsaturated fatty acids in patients with gastric cancer. Therefore, through systematic review, this study analyzed the clinical value of EN preparations containing ω-3 polyunsaturated fatty acids compared with conventional EN preparations in perioperative nutritional support therapy for gastric cancer patients at home and abroad. On the one hand, it provided high-quality grade evidence for clinical nutritional support therapy for gastric cancer patients, and on the other hand, it provided theoretical basis for clinical standardized application of gastric cancer patients. Enteral immunonutrition technology is one of the effective ways to improve this predicament.

## 2 Materials and methods

### 2.1 Literature search strategy

The databases of Pubmed, EMbase, The Cochrane Library, CNKI and Wanfang Medical were searched by computer from the establishment of the database to August 1, 2024. The English search terms included “fatty acids”, “ω-3”, “n-3” and “gastric cancer”, while the Chinese search terms included “enteral immune nutrition”, “polyunsaturated fatty acids”, “ω-3 polyunsaturated fatty acids”, “n-3 polyunsaturated fatty acids” and “gastric cancer”.

### 2.2 Study selection

#### 2.2.1 Inclusion criteria

① The study type was randomized non-placebo controlled trial. ② The study subjects were patients with gastric cancer who received EN containing ω-3 polyunsaturated fatty acids or conventional EN during perioperative period. ③ The outcome measures included safety indicators and efficacy indicators, among which safety indicators included postoperative infection complications; The efficacy indexes included perioperative mortality, immune indexes, inflammatory indexes, and nutritional indexes.

#### 2.2.2 Exclusion criteria

Full text not available; Incomplete data; Single-arm study; Duplicate publications; The surgical site does not meet the requirements; No clinical outcome measures or intermediate measures were reported; Subjective qualitative research.

### 2.3 Data extraction and quality assessment

Contributions were screened independently by two researchers according to inclusion and exclusion criteria. Information, including study author, time, and general characteristics of participants, was extracted using a pre-designed data extraction form and submitted one by one Cross check. If there are differences, they are resolved through discussion. If the data reported in the study is incomplete, further contact the author of the study to obtain; If no relevant data is obtained, the study is excluded.

The Cochrane risk bias assessment tool was used to evaluate literature quality. The tool evaluated risk bias in seven areas, including random sequence generation, assignment hiding, investigator and subject blinding, blind evaluation of study results, outcome data integrity, selective reporting of risk, and other sources of bias. For each indicator, “low bias risk”, “uncertain bias risk” and “high bias risk” are used for judgment (9).

## 3 Statistical analysis

The methods described in the Cochrane Collaborative network meta-analysis guidelines were used for meta-analysis of data using Review Manager 5.4 statistical software. The P-value of the forest map in the meta-analysis was used to detect the differences among different trials. When the P-value was < 0.05, the results were considered to be significantly different. Chi-square test was used to test the heterogeneity of all included studies. If the heterogeneity was small (*P* > 0.1 and I^2^ ≤ 50%), the fixed-effect model was used for meta-analysis. If the heterogeneity is large (*P* ≤ 0.1 and I^2^ > 50%), the random effects model is used for analysis. If the data provided by the trial cannot be meta-analyzed, only descriptive qualitative analysis will be performed. According to the recommendations of the Cochrane Manual for the production of systematic reviews, publication bias was tested by funnel plot.

## 4 Results

### 4.1 Search results

**Figure 1:**
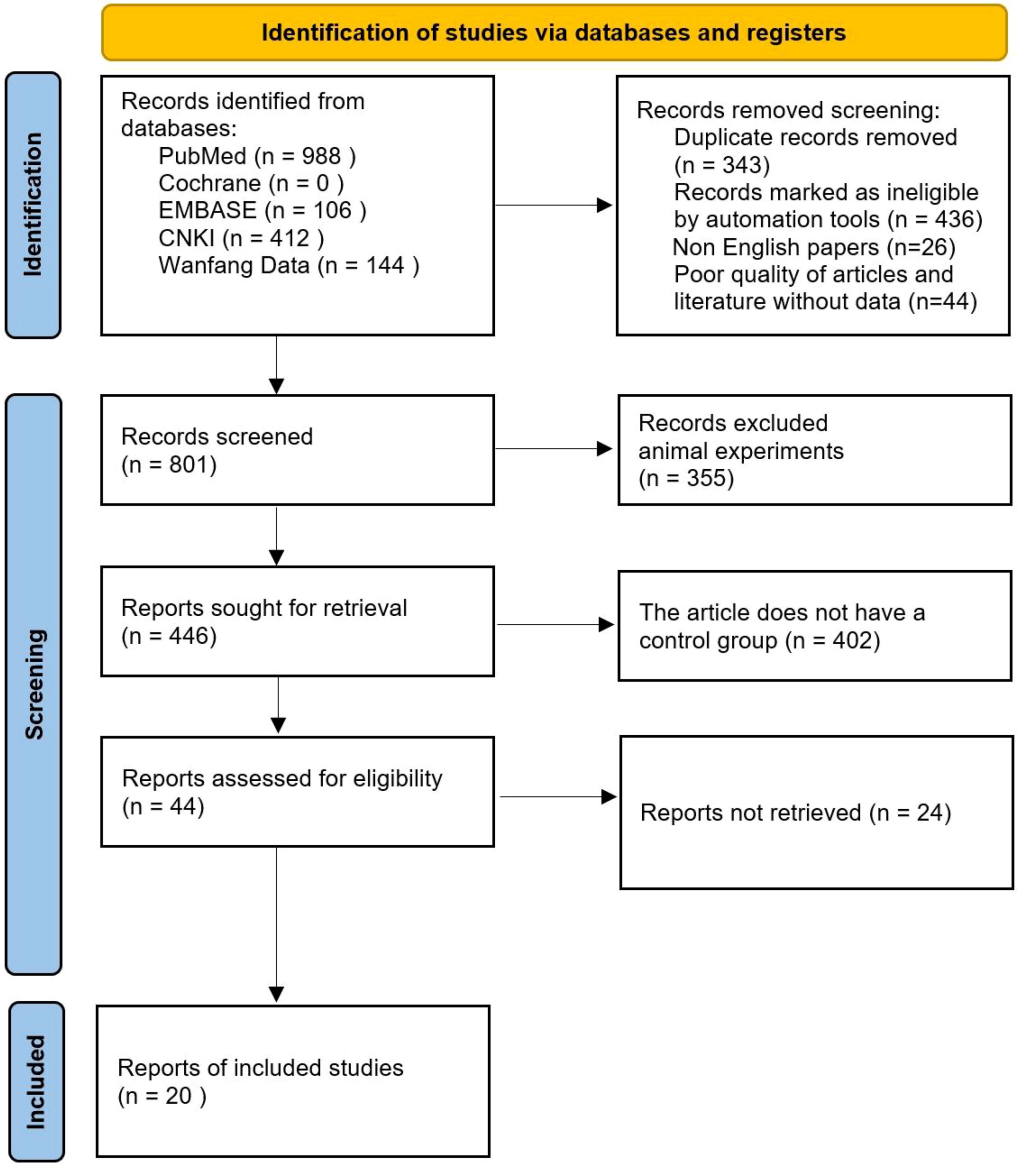
PRISMA Flow chart of article selection.

After initial search, title, abstract preliminary screening, full-text screening and final screening, a total of 20 studies were finally included in the literature search results. Literature search and inclusion process are shown in **Figure 1**.

### 4.2 Study characteristics

**Figure 2:**
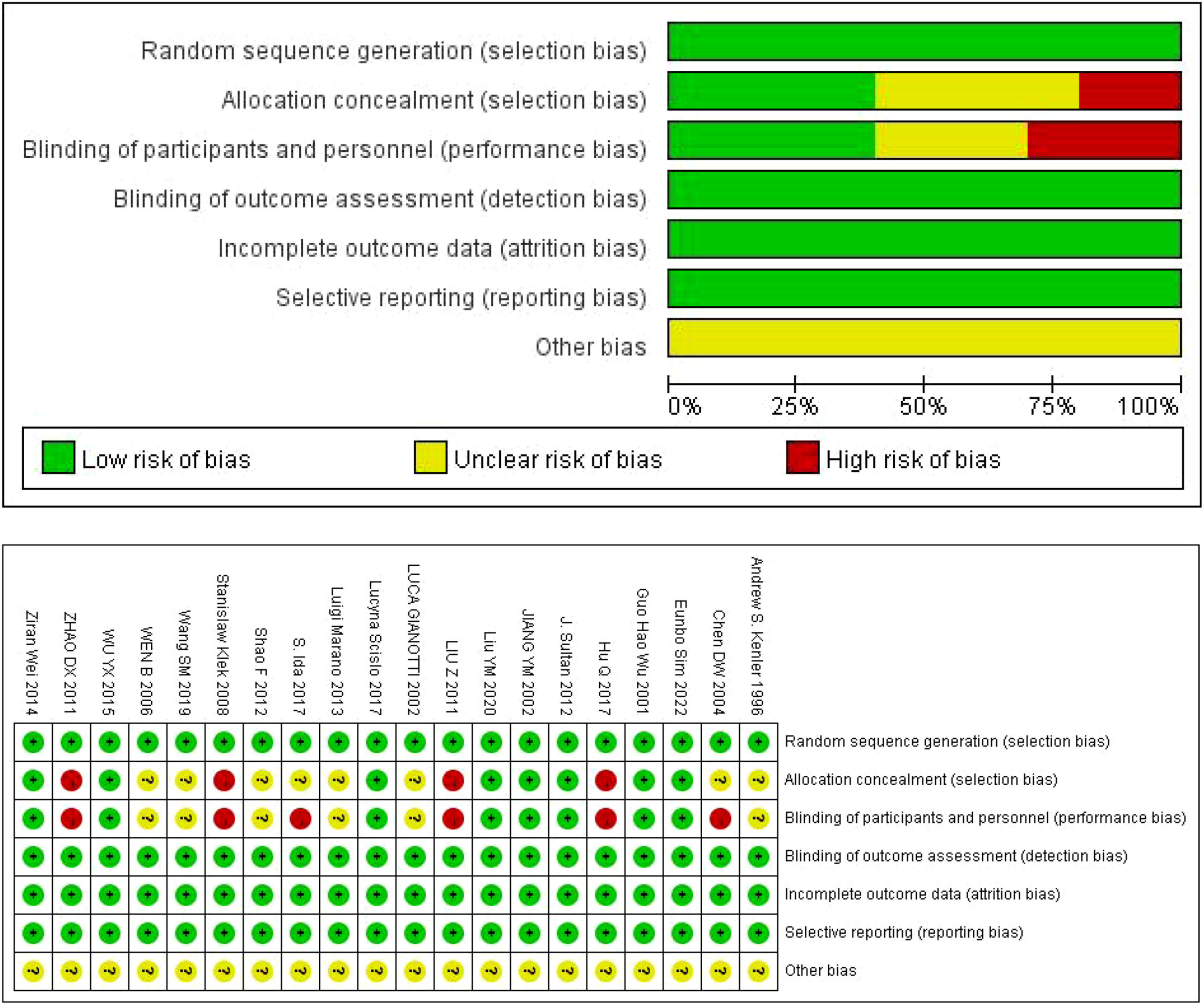
Evaluation results of methodology quality of included studies.

The authors, time, number of cases and other information of the 20 studies included in the characteristics table are shown in **Table 1** (10–29). Of the 20 articles included, 10 were conducted in China. The results of bias risk assessment are shown in **Figure 2**.

**Table 1:** Basic information of literatures (10–29).

Author	Year	Nutritional pathway	Sample	Age	Gender (M/F)
Andrew Skenler (10)	1996	Enteral immunonutrition	17	62.8 ± 2.7	2/15
		Standard enteral nutrition	18	64.4 ± 3.4	7/11
Guo Hao Wu (11)	2001	Enteral immunonutrition	25	55.2 ± 12.1	16/9
		Standard enteral nutrition	23	52.6 ± 9.8	15/8
Luca Gianotti (12)	2002	Enteral immunonutrition	101	58	12/13
		Standard enteral nutrition	102	51	15/11
Jiang YM (13)	2002	Enteral immunonutrition	13	53	14/12
		Standard enteral nutrition	13	53	14/12
Chen DW (14)	2004	Enteral immunonutrition	20	—	—
		Standard enteral nutrition	20	—	—
Wen B (15)	2006	Enteral immunonutrition	39	58.4 ± 12.1	27/12
		Standard enteral nutrition	37	55.5 ± 12.5	27/10
Stanislaw Klek (16)	2008	Enteral immunonutrition	92	62.1	35/13
		Standard enteral nutrition	91	62.3	34/14
Liu Z (17)	2011	Enteral immunonutrition	21	61.10 ± 7.50	15/6
		Standard enteral nutrition	21	61.60 ± 7.20	16/5
Zhao DX (18)	2011	Enteral immunonutrition	21	61.10 ± 7.50	15/6
		Standard enteral nutrition	21	61.60 ± 7.20	16/5
J. Sultan (19)	2012	Enteral immunonutrition	66	67	50/16
		Standard enteral nutrition	63	60	45/18
Shao F (20)	2012	Enteral immunonutrition	33	51	15/17
		Standard enteral nutrition	34	52	17/17
Luigi Marano (21)	2013	Enteral immunonutrition	54	55-78	34/20
		Standard enteral nutrition	55	49-83	37/18
Ziran Wei (22)	2014	Enteral immunonutrition	26	50.5	15/11
		Standard enteral nutrition	20	59	11/9
Wu YX (23)	2015	Enteral immunonutrition	30	52.3 ± 8.6	21/9
		Standard enteral nutrition	30	54.2 ± 11.6	18/12
Lucyna Scislo (24)	2017	Enteral immunonutrition	44	62.6	—
		Standard enteral nutrition	54	62.9	—
S. Ida (25)	2017	Enteral immunonutrition	63	65.1	17/46
		Standard enteral nutrition	60	65.6	17/43
Hu Q (26)	2017	Enteral immunonutrition	36	—	—
		Standard enteral nutrition	36	—	—
Wang SM (27)	2019	Enteral immunonutrition	20	58.0 ± 5.1	9/11
		Standard enteral nutrition	20	59.0 ± 5.4	13/7
Liu YM (28)	2020	Enteral immunonutrition	25	54.6 ± 10.3	18/7
		Standard enteral nutrition	27	56.1 ± 9.8	19/8
Eunbo Sim (29)	2022	Enteral immunonutrition	22	63.64 ± 1.79	—
		Standard enteral nutrition	18	65.39 ± 2.44	—

### 4.3 Meta-analysis outcomes

#### 4.3.1 Mortality rate

**Figure 3:**
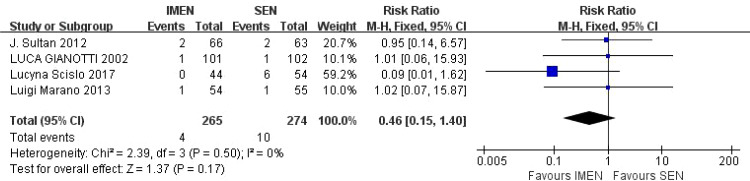
Meta-analysis of mortality rate.

A total of 4 studies reported mortality results, including 265 cases of ω-3 polyunsaturated fatty acids and 274 cases in the control group. I^2^ = 0%, *P* = 0.50 indicated that there was no heterogeneity. Statistical results showed that compared with the control group, the mortality rate in the enteral nutrition with ω-3 polyunsaturated fatty acid preparation group was 0.46 times higher than that in the control group (RR = 0.46, *P* = 0.17), but there was no statistical difference, as shown in **Figure 3**.

#### 4.3.2 Postoperative infectious complications

##### 4.3.2.1 Infectious complications

Results of Infectious Complications were reported in 7 papers, including 362 cases of ω-3 polyunsaturated fatty acids and 361 cases of control. I^2^ = 7%, *P* = 0.37 indicated that there was no heterogeneity. Statistical results showed that compared with the control group, the risk of Infectious Complications in the enteral nutrition with ω-3 polyunsaturated fatty acid preparation group was 0.81 times higher than that in the control group (RR = 0.81, *P* = 0.05), showing a statistical difference, as shown in **Table 2**.

**Table 2:** Meta-analysis of postoperative infectious complications.

Outcomes	Studies	Heterogeneity test results		Effect model	Meta-analysis results	
		*P*	*I^2^*		95%CI	*P*
Infectious Complications	7	0.37	7%	Fixed	0.81 [0.64, 1.03]	0.05
Wound Infection	7	0.83	0%	Fixed	0.61 [0.38, 0.98]	0.04
Respiratory Tract Infection	6	0.91	0%	Fixed	0.81 [0.58, 1.12]	0.20
Urinary Tract Infection	6	0.44	0%	Fixed	1.02 [0.54, 1.94]	0.95
Sepsis	4	0.78	0%	Fixed	0.71 [0.23, 2.21]	0.55
Anastomotic Leakage	4	0.87	0%	Fixed	0.62 [0.29, 1.32]	0.02
Intra-abdominal abscess	4	0.48	0%	Fixed	0.70 [0.30, 1.58]	0.03

##### 4.3.2.2 Wound infection

A total of 7 literatures reported the results of Wound Infection, including 400 cases of ω-3 polyunsaturated fatty acids and 403 cases of control group. I^2^ = 0%, *P* = 0.83 indicated that there was no heterogeneity. Statistical results showed that compared with the control group, the risk of Wound Infection in the enteral nutrition with ω-3 polyunsaturated fatty acid preparation group was 0.61 times that of the control group (RR = 0.61, *P* = 0.04), showing a statistical difference, as shown in **Table 2**.

##### 4.3.2.3 Respiratory tract infection

A total of 6 articles reported the results of Respiratory Tract Infection, including 383 cases of ω-3 polyunsaturated fatty acids and 385 cases of control group. I^2^ = 0%, *P* = 0.91 indicated that there was no heterogeneity. The statistical results indicated that compared to the control group, the risk of Respiratory Tract Infection in the enteral nutrition with ω-3 polyunsaturated fatty acid group is 0.81 times that of the control group (RR = 0.81, *P* = 0.20), but there is no statistical significance. as shown in **Table 2**.

##### 4.3.2.4 Urinary tract infection

A total of 6 literatures reported the results of Urinary Tract Infection, including 339 cases of ω-3 polyunsaturated fatty acids and 331 cases of control group. I^2^ = 0%, *P* = 0.44 indicated that there was no heterogeneity. Statistical results showed that compared with the control group, the risk of Urinary Tract Infection in the enteral nutrition with ω-3 polyunsaturated fatty acid preparation group was marginally elevated by 1.02 times compared to the control group, however, this increase did not reach statistical significance (RR = 1.02, *P* = 0.95), but there was no statistical difference, as shown in **Table 2**.

##### 4.3.2.5 Sepsis

A total of 4 literatures reported Sepsis results, including 238 cases of ω-3 polyunsaturated fatty acids and 238 cases of control group. I^2^ = 0%, *P* = 0.78, indicated that there was no heterogeneity. Statistical results showed that compared with the control group, the risk of Sepsis in the enteral nutrition with ω-3 polyunsaturated fatty acid preparation group is 0.71 times that of the control group (RR = 0.71, *P* = 0.55), but there is no statistical difference, as shown in **Table 2**.

##### 4.3.2.6 Anastomotic leakage

A total of 4 literatures reported the results of Anastomotic Leakage, including 275 cases of ω-3 polyunsaturated fatty acids and 269 cases of control group. I^2^ = 0%, *P* = 0.87 indicated that there was no heterogeneity. The statistical results showed that compared with the control group, the risk of Anastomotic Leakage in the enteral nutrition with ω-3 polyunsaturated fatty acid preparation group was 0.62 times that of the control group (RR = 0.62, P = 0.02), and there was a statistical difference, as shown in **Table 2**.

##### 4.3.2.7 Intra-abdominal abscess

A total of 4 literatures reported the results of Intra-abdominal abscess, including 322 cases of ω-3 polyunsaturated fatty acids and 316 cases of control group. I^2^ = 0%, *P* = 0.48, indicated that there was no heterogeneity. The statistical results showed that compared with the control group, the risk of Intra-abdominal ESS in the enteral nutrition with ω-3 polyunsaturated fatty acid preparation group was 0.70 times higher than that in the control group (RR = 0.70, *P* = 0.03), as shown in **Table 2**.

#### 4.3.3 Postoperative nutritional variables

The results of the meta-analysis indicated that the perioperative nutritional indicators of patients with gastric cancer undergoing surgery in the enteral nutrition with ω-3 polyunsaturated fatty acid preparation group were superior to those in the conventional EN preparation group, specifically for Total protein (WMD = 0.68, *P* = 0.05), Albumin (WMD = 0.34, *P* = 0.03), and Transferrin (WMD = 0.07, *P* = 0.03). However, no statistically significant difference was observed in Pre-albumin (WMD = -0.48, *P* = 0.59). For detailed information, please refer to **Table 3**.

**Table 3:** Meta-analysis of postoperative nutritional variables.

Outcomes	Studies	Heterogeneity test results		Effect model	Meta-analysis results	
		*P*	*I^2^*		95%CI	*P*
Total protein	3	0.54	0%	Fixed	0.68 [-1.36, 2.72]	0.05
Albumin	7	0.95	0%	Fixed	0.34 [-0.35, 1.02]	0.03
Pre-albumin	7	0.0008	74%	Random	-0.48 [-2.22, 1.26]	0.59
Transferrin	7	0.02	59%	Random	0.07 [-0.07, 0.22]	0.03

#### 4.3.4 Postoperative immune indices

The meta-analysis results showed that there was no statistical difference between CD3 (WMD = 4.85, *P* = 0.06) and Total T lymphocyte cells (WMD = -0.17, *P* = 0.66). Perioperative immune indexes of patients with gastric cancer treated with enteral nutrition with ω-3 polyunsaturated fatty acid preparations during surgery (including IgG [WMD = 1.14, *P* < 0.0001], IgA [WMD = 0.36, *P* = 0.0008], IgM [WMD = 0.22, *P* < 0.0001], CD4 [WMD =6.81, *P* = 0.0001] and CD4/CD8 [WMD = 0.46, *P* = 0.002]) were superior to conventional EN preparations. In terms of CD8 (WMD = -1.56, *P* = 0.002), conventional EN preparations were superior to enteral nutrition with ω-3 polyunsaturated fatty acid preparations, and the difference was statistically significant, as shown in **Table 4**.

**Table 4:** Meta-analysis of postoperative immune indices.

Outcomes	Studies	Heterogeneity test results		Effect model	Meta-analysis results	
		*P*	*I^2^*		95%CI	*P*
IgG	6	0.99	0%	Fixed	1.14 [0.83, 1.46]	< 0.0001
IgA	6	0.008	68%	Random	0.36 [0.15, 0.56]	0.0008
IgM	6	0.41	1%	Random	0.22 [0.12, 0.31]	< 0.0001
CD3	4	0.0005	83%	Fixed	4.85 [-0.30, 10.01]	0.06
CD4	8	< 0.00001	84%	Random	6.81 [3.33, 10.29]	0.0001
CD8	8	0.61	0%	Random	-1.56 [-2.57, -0.56]	0.002
CD4/CD8	9	< 0.00001	90%	Fixed	0.46 [0.16, 0.76]	0.002
Total T lymphocyte cells	5	< 0.00001	100%	Random	-0.17 [-0.95, 0.60]	0.66

#### 4.3.5 Postoperative inflammatory indices

The Meta analysis results showed that, except for the results of WBC (WMD = 0.68, *P* = 0.59) and TNF-α (WMD = -11.03, *P* = 0.46), the perioperative inflammatory indexes [including CRP(WMD = -1.51, *P* = 0.04), IL-1β (WMD = -26.34, *P* = 0.0005), IL-6 (WMD =- 5.38, *P* < 0.00001)] of gastric cancer patients treated with enteral nutrition with ω-3 polyunsaturated fatty acid preparations during surgery were better than those of conventional EN preparations, and the difference was statistically significant, as shown in **Table 5**.

**Table 5:** Meta-analysis of postoperative inflammatory indices.

Outcomes	Studies	Heterogeneity test results		Effect model	Meta-analysis results	
		*P*	*I^2^*		95%CI	*P*
WBC	3	< 0.00001	93%	Random	0.68 [-1.78, 3.14]	0.59
CRP	3	0.07	61%	Random	-1.51 [-3.09, 0.08]	0.04
IL-1β	3	0.26	27%	Fixed	-26.34 [-41.25, -11.42]	0.0005
IL-6	6	< 0.00001	99%	Random	-5.38 [-7.03, -3.72]	< 0.00001
TNF-α	4	< 0.00001	99%	Random	-11.03 [-40.16, 18.10]	0.46

### 4.4 Risk of bias

**Figure 4:**
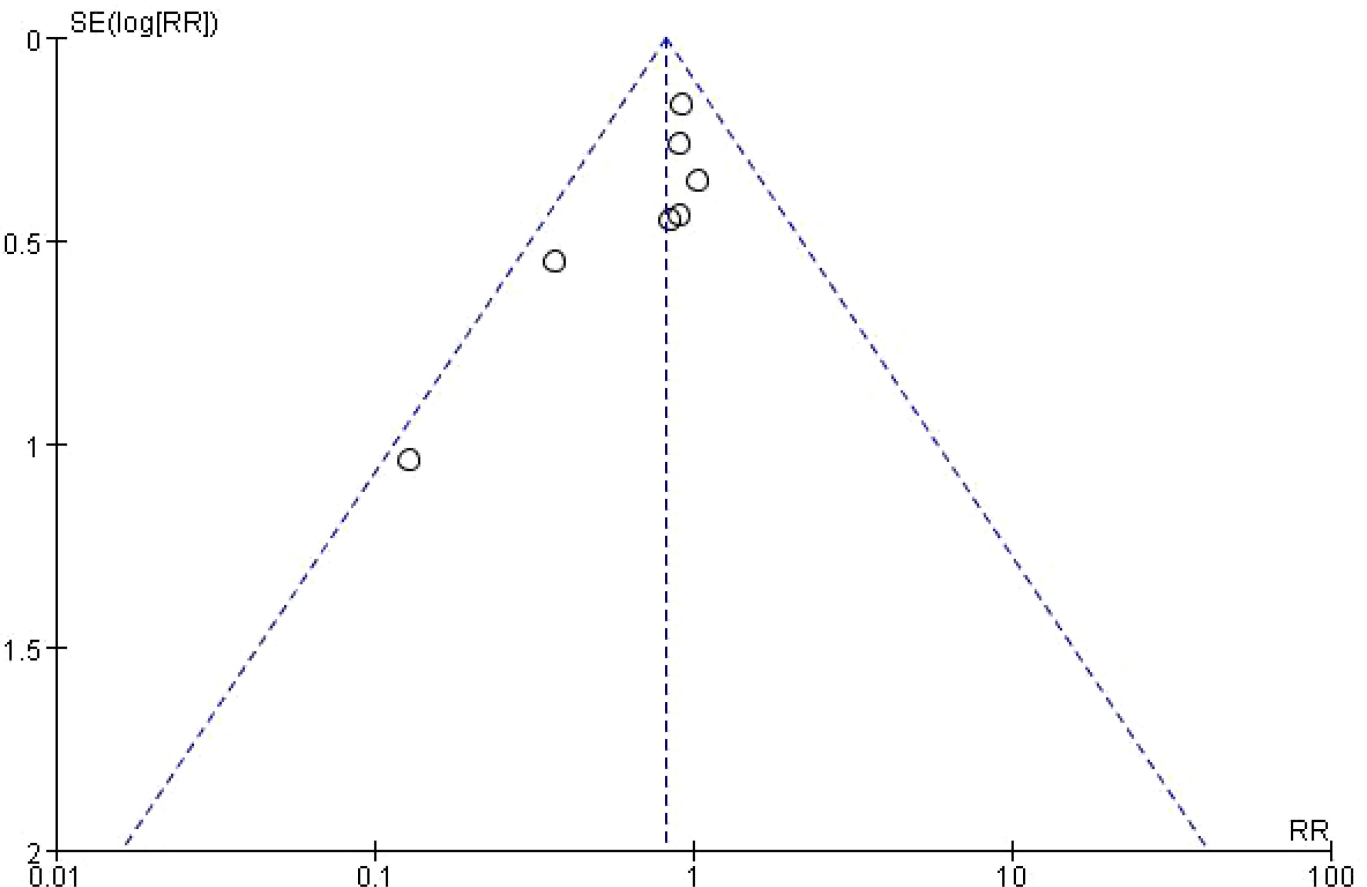
Funnel analysis of Infectious Complications.

Funnel plot was used to analyze the effect of publication bias on meta-analysis results. Taking Infectious Complications as an example, it was shown that the included studies were evenly distributed on both sides of the funnel plot, suggesting that publication bias had little effect on the results in this study, as shown in **Figure 4**.

## 5 Discussion

Malnutrition is a clinical condition in which the lack of energy, protein and other nutrients in the body leads to adverse outcomes in the body and in the clinic. With the improvement of people’s living standards, the probability of malnutrition in normal adults has decreased significantly compared with the past. In recent years, the incidence of malignant tumors has increased year by year, the proportion of nutritional risk in patients has exceeded 50%, and the incidence of malnutrition has reached over 30% (30–32). Studies have shown that systemic inflammation is common in patients with malignant tumors. Systemic inflammation occurs at the early stage of the tumor and worsens with the progression of the disease. Inflammation promotes tumor progression through multiple ways and affects nutrient metabolism, resulting in an imbalance in energy intake and utilization (33–36). Dewys et al. (37) reported that 87% of gastric cancer patients were undernourished, and the incidence of cachexia was as high as 65%-85%, ranking first among malignant tumors. Clinical evidence shows that the occurrence of malnutrition in tumor patients has a certain impact on chemotherapy efficacy and tolerance to chemotherapy drugs (38–41). Therefore, in order to ensure the therapeutic effect of malignant tumors, it is necessary to take nutritional support therapy for patients to improve body function, improve clinical efficacy, and reduce the incidence of complications and mortality.

The early diagnosis rate of early gastric cancer is low due to its inconspicuous clinical manifestations or non-specific characteristics, and most patients are already in the advanced stage when diagnosed. Clinical data show that patients with advanced gastric cancer are often accompanied by severe malnutrition, which is mainly caused by the following three factors (42): (1) anorexia and fullness caused by tumor; (2) Mechanical obstruction of digestive tract caused by protrusion of cancer; (3) Adverse reactions occurred during tumor treatment, such as gastrointestinal mucosal injury and abnormal secretion of digestive fluid. Patients with advanced gastric cancer often lose the chance of surgery, and chemotherapy is an important treatment method for them, while the incidence of malnutrition in patients with advanced gastric cancer is 15%-55% (43). Studies have reported that patients with advanced gastric cancer accompanied by malnutrition have a significant decline in chemotherapy tolerance, and it is difficult to complete chemotherapy as planned (24).Therefore, for advanced gastric cancer patients with malnutrition, their nutritional status should be improved at the same time of chemotherapy to eliminate the negative effects of malnutrition as far as possible.

The commonly used nutritional support methods in clinic include enteral nutrition and parenteral nutrition. Compared with parenteral nutrition, enteral nutrition can not only supplement the nutrients required by the body, but also protect the integrity of the intestinal mucosal barrier structure and reduce the damage to gastrointestinal function. Enteral immune nutrition preparation is a method used in clinical application in recent years to improve the immune function of patients. It refers to adding glutamine, arginine, ω-3 unsaturated fatty acids, nucleotides and other immunomodulatory nutrients to the standard enteral nutrition formula. These immunomodulatory components can stimulate the immune cells of the body and improve the immunity of the body after being absorbed by the body. ω-3 polyunsaturated fatty acids mainly include α-linolenic acid, eicosapentaenoic acid and docosahexaenoic acid. Studies have shown that ω-3 polyunsaturated fatty acids can play a role in mediating the inflammatory response of patients with malignant tumors by changing the structure, function and fluidity of cell membranes and reducing the production of pro-inflammatory mediators through competitive inhibition (7). *In vitro* cell tests showed that the secretion levels of pro-inflammatory factors TNF-α and IL-6 in macrophages were changed after the intervention of ω-3 polyunsaturated fatty acids, which further confirmed the direct anti-inflammatory effect of ω-3 polyunsaturated fatty acids on macrophages (44). Numerous clinical studies have shown that ω-3 polyunsaturated fatty acids, as an immune nutrient, play an important pharmacological role in the prevention and treatment of diabetes, hypertension, arthritis, tumor and other inflammatory and autoimmune diseases (45).

The research on the relationship between ω-3 unsaturated fatty acids and tumor prevention began 30 years ago, but there is still controversy to this day. Brasky TM et al. (46) believed that dietary supplementation of ω-3 unsaturated fatty acids could reduce the risk of breast cancer (HR = 0.68, 95%CI = 0.50-0.92). In 1982, Chavarro JE et al. tracked the blood levels of ω-3 unsaturated fatty acids in 14916 healthy men for a period of 13 years. Finally, 476 subjects were diagnosed with prostate cancer and matched with a control group. It was found that those with lower blood levels of ω-3 unsaturated fatty acids had an increased risk of developing prostate cancer (RR = 0.59, 95%CI = 0.38-0.93) (47). Hall MN et al. tracked the concentration of blood ω-3 unsaturated fatty acids in 21406 healthy individuals for a period of 22 years. Finally, 500 cases were diagnosed with colorectal cancer (388 cases of colon cancer and 112 cases of rectal cancer). They believed that as the intake of ω-3 unsaturated fatty acids increased, the risk of colorectal cancer decreased (RR = 0.74, 95%CI = 0.57-0.95) (48). The possible mechanism of ω-3 unsaturated fatty acids on tumor development is related to their function in regulating inflammatory response. Inflammatory response plays an important role in the occurrence and progression of tumors, so a diet rich in ω-3 unsaturated fatty acids A may have a preventive effect on tumors. Omega-6 unsaturated fatty acids are another type of polyunsaturated fatty acid in the human body. The first unsaturated bond in their molecule appears at the sixth position of the methyl end of the carbon chain, including linoleic acid and arachidonic acid. ω-3 unsaturated fatty acids and omega-6 unsaturated fatty acids share the same metabolic enzymes, namely cyclooxygenase and lipoxygenase. EPA and AA can both produce inflammatory mediators through the action of cyclooxygenase and lipoxygenase, but the pro-inflammatory and pro proliferative activity caused by EPA’s production of inflammatory mediators is relatively weak (49). ω-3 unsaturated fatty acids have a stronger affinity for this type of enzyme and can competitively inhibit the conversion of ω-6 unsaturated fatty acids, reducing the synthesis of arachidonic acid and thus alleviating inflammatory reactions. Therefore, a diet rich in ω-3 unsaturated fatty acids can negatively regulate the inflammatory cascade response and reduce the risk of tumor development (50).

This study investigated the therapeutic efficacy of enteral nutrition with ω-3 polyunsaturated fatty acid preparations versus standard enteral nutrition (SEN) emulsions in perioperative management of patients with advanced gastric cancer experiencing malnutrition. Although no statistically significant disparity was noted in mortality rates between the two groups, notable differences emerged in indicators pertaining to infection, immunity, inflammation, and nutrition. Specifically, the incidence of infection-related complications, such as wound infections, abdominal abscesses, and stoma complications, was significantly lower in the IEN group compared to the SEN group. Furthermore, the IEN group demonstrated enhanced recovery of immune markers (IgG, IgA, IgM, CD4, and CD4/CD8 ratios) and nutritional indices (Total protein, Albumin, and Transferrin), suggesting a positive impact on overall immune status and nutritional replenishment. Regarding inflammation, the IEN group exhibited reduced levels of CRP, IL-1β, and IL-6, indicating an attenuation of the inflammatory response compared to the SEN group. These findings underscore the significant value of enteral nutrition with ω-3 polyunsaturated fatty acid in ameliorating perioperative nutritional status and potentially improving clinical outcomes for patients with advanced gastric cancer. The results of this study are also consistent with the aforementioned research findings.

## 6 Conclusion

In summary, enteral nutrition preparations containing ω-3 polyunsaturated fatty acids have more advantages than standard nutrition preparations in the perioperative nutritional treatment of gastric cancer patients in terms of effectiveness and safety, and can be used as the preferred clinical drug. Due to the limited number and quality of included studies, the above conclusions need to be verified by more high-quality studies.

## Data Availability

The original contributions presented in the study are included in the article/supplementary material, further inquiries can be directed to the corresponding author/s.
